# Mutation Analysis of the *ERCC4/FANCQ* Gene in Hereditary Breast Cancer

**DOI:** 10.1371/journal.pone.0085334

**Published:** 2014-01-21

**Authors:** Sandra Kohlhase, Natalia V. Bogdanova, Peter Schürmann, Marina Bermisheva, Elza Khusnutdinova, Natalia Antonenkova, Tjoung-Won Park-Simon, Peter Hillemanns, Andreas Meyer, Hans Christiansen, Detlev Schindler, Thilo Dörk

**Affiliations:** 1 Clinics of Obstetrics and Gynaecology, Hannover Medical School, Hannover, Germany; 2 Clinics of Radiation Oncology, Hannover Medical School, Hannover, Germany; 3 Institute of Biochemistry and Genetics, Ufa, Russia; 4 N.N. Alexandrov Research Institute of Oncology and Medical Radiology, Minsk, Belarus; 5 Institute of Human Genetics, Biocenter, University of Würzburg, Würzburg, Germany; Cancer Research Centre of Lyon, France

## Abstract

The ERCC4 protein forms a structure-specific endonuclease involved in the DNA damage response. Different cancer syndromes such as a subtype of Xeroderma pigmentosum, XPF, and recently a subtype of Fanconi Anemia, FA-Q, have been attributed to biallelic *ERCC4* gene mutations. To investigate whether monoallelic *ERCC4* gene defects play some role in the inherited component of breast cancer susceptibility, we sequenced the whole *ERCC4* coding region and flanking untranslated portions in a series of 101 Byelorussian and German breast cancer patients selected for familial disease (set 1, n = 63) or for the presence of the rs1800067 risk haplotype (set 2, n = 38). This study confirmed six known and one novel exonic variants, including four missense substitutions but no truncating mutation. Missense substitution p.R415Q (rs1800067), a previously postulated breast cancer susceptibility allele, was subsequently screened for in a total of 3,698 breast cancer cases and 2,868 controls from Germany, Belarus or Russia. The Gln415 allele appeared protective against breast cancer in the German series, with the strongest effect for ductal histology (OR 0.67; 95%CI 0.49; 0.92; p = 0.003), but this association was not confirmed in the other two series, with the combined analysis yielding an overall Mantel-Haenszel OR of 0.94 (95% CI 0.81; 1.08). There was no significant effect of p.R415Q on breast cancer survival in the German patient series. The other three detected *ERCC4* missense mutations included two known rare variants as well as a novel substitution, p.E17V, that we identified on a p.R415Q haplotype background. The p.E17V mutation is predicted to be probably damaging but was present in just one heterozygous patient. We conclude that the contribution of *ERCC4/FANCQ* coding mutations to hereditary breast cancer in Central and Eastern Europe is likely to be small.

## Introduction

An individual's breast cancer risk is shaped by inherited variation in genes that normally ensure genome stability through their function in DNA damage recognition and repair. This includes the resolution of deleterious DNA structures that result as a consequence of damage or from abnormal replication and recombination intermediates. The proteins ERCC4 (also known as XPF or FANCQ) and ERCC1 form a heterodimeric structure-specific endonuclease that promotes DNA cleavage at single-stranded/double-stranded junctions as a prerequisite to the removal of structures resulting from DNA intra- and interstrand crosslinks [1]–[3]. The ERCC1/ERCC4 complex is recruited by the XPA protein to sites of nucleotide excision repair [4]. ERCC4 activity is regulated by SLX4, a coordinator of structure-specific endonucleases required for the resolution of Holliday junctions and interstrand crosslink repair [5]. ERCC4 also interacts with RAD52, and the binding of RAD52 and ERCC4 concomitantly stimulates the endonuclease activity of ERCC1/ERCC4 and attenuates the DNA strand annealing activity of RAD52 during homologous recombination [6]. Thus, ERCC4 is a versatile protein that is required for different types of DNA repair.

The *ERCC4* gene is located on chromosome 16p13.12, consists of eleven exons and encodes a 104 kDa protein of 916 amino acids. Defects of *ERCC4* are the cause of xeroderma pigmentosum complementation group F (XP-F) [MIM:278760], and the XFE progeroid syndrome (XFEPS) [MIM:610965] which includes stunted growth and microcephaly. More recently, biallelic mutations of the *ERCC4* gene have been identified as the cause of Fanconi Anemia type Q and Cockayne syndrome [7], [8]. Fanconi Anemia (FA) is a rare recessive disorder characterized by congenital malformations, progressive bone marrow failure and predisposition to cancer. Sixteen different FA genes have now been identified whose products act in a common pathway of DNA interstrand crosslink repair, and some of them (including *BRCA2/FANCD1*, *BRIP1/FANCJ*, *PALB2/FANCN*, and *RAD51C/FANCO*) have also been described as breast and ovarian cancer susceptibility genes [9], [10]. It is still unclear, however, whether *ERCC4* gene alterations also play some role in the inherited component of breast cancer susceptibility. In the present study, we investigated the mutational spectrum of the *ERCC4/FANCQ* coding sequence in a series of German or Byelorussian patients with familial breast cancer.

## Results

The whole coding region and flanking sequences were analysed by direct sequencing of the 11 exons of the *ERCC4* gene in genomic DNA samples from 25 German and 38 Byelorussian breast cancer patients with a family history of disease (Set 1). We confirmed six known single nucleotide polymorphisms (SNPs) in both non-coding and coding sequences, including the missense substitution p.R415Q, and three rare exonic variants, including the missense substitution p.I73V (Table 1). None of the substitutions was predicted to affect splicing as judged by MaxEntScan. Of the two missense substitutions, the p.R415Q was predicted to be deleterious by CONDEL (Consensus Deleterious Score 0.905).

**Table 1:** Genetic alterations of the *ERCC4* gene in German and Byelorussian breast cancer patients.

Location	Nucleotide change	Codon	No. of carriers (frequency)			NCBI database annotation
			het	hom	total	
*Variants in patients with familial breast cancer (Set 1)*						
Upstream	c.−30T>A	none	22 (.35)	3 (.05)	25 (.40)	rs1799797
Exon 1	c.33C>T	p.A11A	2 (.03)	-	2 (.03)	rs3136042
Intron 1	c.207+11G>A	none	22 (.35)	3 (.05)	25 (.40)	rs762521
Intron 1	c.207+49G>A	none	9 (.14)	-	9 (.14)	rs1799798
Exon 2	c.217A>G	p.I73V	1 (.02)	.	1 (.02)	rs141591400
Exon 8	c.1244G>A	p.R415Q	8 (.13)	2 (.03)	10 (.16)	rs1800067
Intron 9	c.1905−35T>C	none	24 (.38)	5 (.08)	29 (.46)	rs1799799
Exon 11	c.2505T>C	p.S835S	20 (.32)	3 (.05)	23 (.37)	rs1799801
Exon 11	c.2655G>A	p.T885T	1 (.02)	-	1 (.02)	rs16963255
*Additional variants identified in p.R415Q carriers (Set 2)*						
Exon 1	c.50A>T	p.E17V	1 (.01)*	-	1 (.01)*	not listed
Exon 11	c.2624A>G	p.E875G	2 (.02)*	-	2 (.02)*	rs1800124

Survey of genetic alterations of the *ERCC4* gene identified in a sequencing study of 101 patients selected for familial breast cancer (n = 63, Set 1) or the p.R415Q haplotype (n = 38, Set 2). Mutations were designated according to the improved mutation nomenclature recommended by the Human Genome Variation Society [www.hgvs.org/mutnomen/], using the Ensembl transcript ID ENST00000311895 and protein ID ENSP00000310520 for the ERCC4 coding sequence. Rs numbers refer to the NCBI SNP database [http://www.ncbi.nlm.nih.gov/sites/entrez]. Het, heterozygous; hom, homozygous.

* Percentage refers to the total series of 101 sequenced patients.

Since the p.R415Q substitution turned out to be more common and had been reported as a potential risk allele [30], [31], it was tested for its association with breast cancer in three independent case-control studies from Germany, Belarus and Russia. The p.R415Q allele tended to associate with breast cancer in the HaBCS series, though with a protective effect of the rare allele (Table 2). The allelic effect was restricted to ductal breast cancer (OR 0.67, 95%CI 0.49;0.92; p = 0.003) and was also apparent in patients with a positive family history (p = 0.02), with high-grade tumours (p = 0.02) and with positive nodal status (p = 0.04)(Table 2). However, none of these findings could be replicated in the other two studies, HMBCS and HUBCS, and the combined analysis did not confirm a significant association (Mantel-Haenszel OR 0.94, 95%CI 0.82; 1.08) (Table 2). Because some SNPs in double-strand break repair genes may also impinge on prognosis, we further assessed whether the ERCC4*p.R415Q substitution could affect survival from breast cancer. In the 829 patients for whom follow-up data were available, the presence of the p.R415Q allele had no detectable effect on the breast cancer specific survival (HR 0.53; 95% CI 0.26–1.10; p = 0.62) nor on the overall survival (HR 0.63; 95% CI 0.34–1.17; p = 0.34).

**Table 2:** Association study of the *ERCC4* missense variant p.R415Q in three breast cancer case-control series.

Study	*ERCC4* *p.R415Q genotypes (RR/RQ/QQ)		per allele OR (95% CI)	p_trend_
	cases	controls		
HaBCS (Germany)	808/123/1	827/151/5	0.81 (0.63; 1.03)	0.07
- ductal	529/65/0	.	0.65 (0.48; 0.87)	0.003**
- FH+ve	132/12/0	.	0.50 (0.27; 0.91)	0.02*
- ER+ve	496/74/0	.	0.78 (0.58; 1.04)	0.08
- high grade	265/31/0	.	0.62 (0.42; 0.92)	0.02*
- node+ve	225/28/0	.	0.66 (0.43; 0.99)	0.04*
HMBCS (Belarus)	1721/205/11	1086/141/2	0.99 (0.80; 1.23)	0.95
- ductal	473/60/3	.	1.05 (0.76; 1.41)	0.77
- FH+ve	268/34/3	.	0.98 (0.66; 1.45)	0.54
- ER+ve	853/90/4	.	0.87 (0.67; 1.13)	0.30
- high grade	154/20/1	.	1.07 (0.67; 1.70)	0.77
HUBCS (Russia)	748/75/6	593/62/1	1.08 (0.78; 1.50)	0.66
- ductal	291/24/2	.	0.90 (0.57; 1.42)	0.66
- FH+ve	24/2/0	.	0.78 (0.19; 3.28)	0.73
- high grade	60/8/0	.	1.21 (0.57; 2.59)	0.61
- node+ve	241/19/0	.	0.74 (0.44; 1.24)	0.25
**Total**	**3277/403/18**	**2506/354/8**	**0.94 (0.82; 1.08)**	**0.39**

Results from an association study of the missense substitution p.R415Q in case-control series from Germany (HaBCS: 932 cases/983 controls), Belarus (HMBCS: 1,937 cases/1,229 controls) and Russia (HUBCS: 829 cases/656 controls). Minor allele frequency (MAF) in controls was 0.08 in HaBCS and 0.06 in HMBCS, and was 0.06 for each of both, Russians and Tatars in HUBCS but was lower in the Bashkir subpopulation (MAF 0.01). Per-allele odds ratios were calculated in comparison to the controls for all breast cancer patients and for specific subgroups after stratification by ductal histology, 1° degree family history of breast cancer, tumour grade (high grade defined as >2) and – where available - estrogen receptor status and nodal status. P_trend_ values were calculated under an additive model using Armitage trend tests. For the total, Mantel-Haenszel Odds Ratio and p-value were obtained from a combined analysis with stratification by study population.

* p<0.05;

** p-value for ductal histology remained significant after Bonferroni correction.

**Figure 1:**
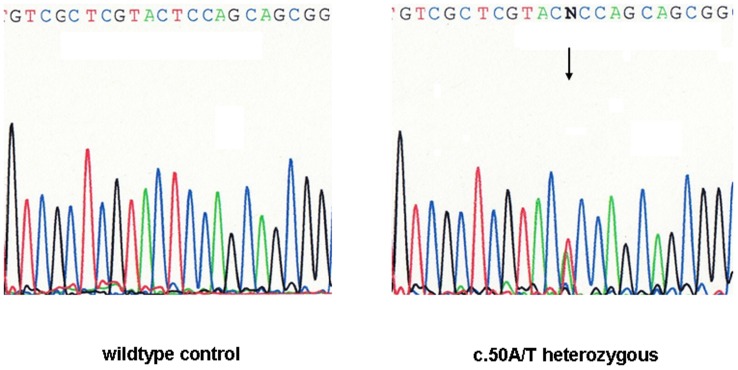
Identification of a novel missense mutation in the *ERCC4* gene. Identification of the missense mutation p.E17V (c.50A>T) in the *ERCC4* gene by direct sequencing (reverse strand). Left panel: wildtype, right panel: heterozygous carrier of p.E17V.

To investigate whether the apparent differences in risk between the studies could be attributed to second-site mutations in linkage disequilibrium with p.R415Q, we additionally sequenced the whole *ERCC4* coding region in 12 homozygotes and 26 heterozygotes (Set 2), thus representing 50 p.R415Q alleles identified during the previous screening. This approach uncovered two additional missense variants, including a novel p.E17V substitution (Figure 1). The substitution p.E17V is a non-conservative missense substitution that is predicted by CONDEL to be deleterious with a score of 0.903. It is presently unconfirmed whether this missense variant is indeed pathogenic, as only one heterozygous patient was identified. The patient who was a homozygote for p.R415Q, originated from Belarus and had been diagnosed with an ER-positive lobular breast cancer at the age of 42 years, but had no family history of the disease. The other missense substitution was a p.E875G substitution that occurred in the heterozygous state in two unrelated German patients with bilateral breast cancer but is known as a rare variant (c.2624A>G, rs1800124) that, although located in the evolutionary conserved ruvA-like domain, is predicted to be neutral by CONDEL. The paucity of mutations in the other carriers indicates that any effect of the p.R415Q allele is unlikely to be explained by second-site mutations in the *ERCC4* coding region.

## Discussion

Functional deficiencies in DNA repair are a hallmark of genomic instability syndromes as well as of mammary carcinogenesis. Germline mutations in at least four Fanconi Anemia genes (*BRCA2, PALB2, RAD51C, BRIP1*) have thus far been found to contribute to the inherited risk of breast or ovarian cancer [9], [10]. The ERCC4 protein is also required for the repair of DNA interstrand crosslinks and has recently been identified as another component of the Fanconi Anemia pathway and novel Fanconi Anemia protein [7], [8]. *ERCC4* is therefore a plausible candidate gene to investigate whether inactivating germ-line mutations in its coding sequence may also contribute to breast cancer risk. Furthermore a missense polymorphism, p.R415Q, has been associated with benign breast disease, a potential intermediate marker of breast cancer risk, and with an increased breast cancer risk in Europeans [30], [31]. The presence of *ERCC4* mutations may also be of therapeutic relevance as inhibitors that target the ERCC1-XPF complex have been proposed as a novel strategy to overcome chemoresistance towards cross-linking agents [32]. In the present work, we have sequenced all exons and flanking non-coding sequences of *ERCC4* in a total of 75 breast cancer patients. This selected series included an exploratory set of 25 German and 38 Byelorussian patients with a pronounced family history of breast cancer, and a subsequent validation set of 38 breast cancer patients with a homozygous or heterozygous p.R415Q background.

The two coding variants that were found in the first set of patients were p.I73V and p.R415Q. The substitution p.I73V is a conservative missense variant that has been listed in the 1000genomes database with a minor allele frequency of 0.001 in Europeans and is predicted to be neutral. There is little evidence to postulate any effect for this variant. More common is p.R415Q, a known missense polymorphism (rs1800067) encoding a non-conservative change from arginine to glutamine that is predicted to be deleterious. This polymorphism has been associated with benign breast disease [30], and a meta-analysis of seven different studies comprising 3,910 cases and 3,985 controls had previously suggested that Arg415Gln may be a low-penetrant risk factor in the Caucasian ethnicity for developing breast cancer [31]. However, in our screening of three different populations, the p.R415Q allele tended to associate with breast cancer in only one study, HaBCS, where the association was restricted to ductal breast cancer and was in the opposite direction as the risk effect reported by Ding and co-workers [31]. Since our study has a total size that is similar to the previously published meta-analysis, we conclude that the p.R415Q is unlikely to exert a strong effect on breast cancer risk. Similar conclusions have been drawn for another SNP (rs744154) in intron 1 of *ERCC4* that is weakly correlated with p.R415Q and failed to be reproduced in larger studies [23], [33].

Additional *ERCC4* mutations are unlikely to account for study heterogeneity, since our resequencing of 12 p.R415Q homozygotes and 26 p.R415Q heterozygotes led to the detection of two further potential mutations (p.E17V, p.E875G) in three patients, thereby representing at most some 6% of the sequenced p.R415Q chromosomes. The substitution p.E17V is a non-conservative missense substitution that is predicted to be deleterious. The substitution p.E875G is a non-conservative missense substitution in a conserved domain but is predicted to be neutral. It is presently undetermined whether these variants, alone or in combination with p.R415Q, would indeed be pathogenic, as the heterozygous patients identified had no strong family history of cancer. For p.E875G, that is listed with an allele frequency of 0.009 (rs1800124), much larger case-control studies would be required to clarify any risk association. For p.E17V, that is neither listed in the 1000genomes project nor in the NCBI SNP database and appears to be a very rare variant, the potential role of the p.E17V-p.R415Q double substitution for breast cancer risk, if any, is expected to be limited.

While this manuscript was under review, an independent study was published that assessed the frequency of *ERCC4* mutations in Spanish breast cancer patients [34]. Osorio *et al.* scanned 1,573 breast cancer patients using DHPLC and identified one truncating mutation and two functionally relevant missense variants. Their mutation frequency in cases was not different from controls, suggesting that *ERCC4* is not a breast cancer susceptibility gene [34]. Missense substitution p.R415Q was not addressed specifically, but as this allele is not uncommon in Spain, mutations associated with it would have been picked up. Thus, our observations are in full concordance with results from the Spanish population.

Altogether, this study did not support a major role for *ERCC4* coding variants in familial breast cancer risk, although rare mutations such as p.E17V could make minor contributions. We also did not confirm previously suggested associations of the p.R415Q substitution with an increased breast cancer risk. The rarity of pathogenic mutations in our study seems to be consistent with the view that germ-line mutations in the *ERCC4/FANCQ* gene might account for only a very small proportion of breast cancer patients in Central and Eastern Europe.

## Methods

### Patients

Our study group consisted of two large breast cancer case-control series, the Hannover Breast Cancer Study (HaBCS) and the Hannover-Minsk Breast Cancer Study (HMBCS). In addition, the Hannover-Ufa Breast Cancer Study (HUBCS), served as a further validation set for the p.R415Q screening.

The HaBCS includes a hospital-based series of 1012 unselected German breast cancer patients who were treated at the Department of Radiation Oncology at Hannover Medical School from 1996–2001. This patient series had been used previously to determine the frequency and risks of intermediate-penetrance mutations [11]–[18], and to study some more common polymorphisms in candidate genes by the Breast Cancer Association Consortium [19]–[24]. Genomic DNA was available for 965 patients, and follow-up data were available for 829 patients, with a median follow-up time of 7.6 years. 34 patients were eligible for the *ERCC4* resequencing study on the basis of a family history score of at least 1.5 (corresponding to at least one first and one second degree relative with breast cancer). Of those, seven known carriers of a *BRCA1* or *BRCA2* mutation and two samples with small amounts of DNA were excluded. Thus, there remained 25 patients for the initial *ERCC4* sequencing study. Population controls were randomly taken from a cohort of healthy female German blood donors recruited in 2005 at the same university hospital.

The HMBCS has also been described previously and has been subject of genetic association studies of rare susceptibility alleles [14]–[16], [18], [25], [26] or common polymorphisms [22], [24], [27]. Byelorussian cases were 1945 female breast cancer patients who had been diagnosed during the years 1998–2008 at the Byelorussian Institute for Oncology and Medical Radiology Aleksandrov N.N. in Minsk or at one of the regional oncology centers in Gomel, Mogilev, Grodno, Brest or Vitebsk, respectively. Family history was available for 97.2% of the cases. Of those, we singled out 40 patients who had a family history score of at least 2 (corresponding to two first degree relatives, or one first and two second degree relatives, or one first and one second and two third-degree relatives with breast cancer). Two patients who were known carriers of a *BRCA1* mutation were excluded. Thus, we remained with 38 patients for the initial *ERCC4* sequencing study. Population controls were taken from a cohort of healthy female Byelorussian blood donors who had no personal or family history of cancer and were recruited at the Minsk center during the same time period.

The HUBCS series has also been described previously [18], [25]. This series from Russia consisted of 1,059 breast cancer patients unselected for family history who had been diagnosed during the years 2000–2007 at the oncological center in Ufa (Bashkortostan). Healthy population controls included 1,069 volunteers from the same geographic regions, with a similar ethnic distribution. This series was used for TaqMan screening only, with a preference given to samples of mainly European descent.

### Mutation analyses

Genomic DNA was isolated from peripheral EDTA blood samples using standard phenol-chloroform extraction. All coding exons of the *ERCC4* gene were amplified by polymerase chain reaction using previously reported primer pairs with sequences flanking the respective exons [7]. 35 cycles of PCR were carried out using HotStart Taq DNA Polymerase (Qiagen) with 1 min annealing at the primer specific temperature, 1 min extension at 72°C and 1 min denaturation at 94°C. Sequencing reactions were performed using one of the PCR primers with BigDye v1.1 chemistry, and sequences were evaluated on a Genetic Analyzer 3100 Avant (Applied Biosystems). The missense substitution p.R415Q was subsequently screened for in three case-control series using a commercially available TaqMan 5′-exonuclease assay (C___3285104_10, Applied Biosystems) on a 7500 FAST Genetic Analyzer (Applied Biosystems). Call rates were above 95% for each study.

### Statistical analyses

Genotype data and allele frequencies for missense variant p.R415Q were tested for consistency with Hardy Weinberg equilibrium and odds ratios were estimated under additive, dominant and recessive models using SNP & Variation Suite 7 Software (Golden Helix Inc.). Allele and genotype frequencies were compared using chi-square tests, or an Armitage trend test in the additive model. These analyses were performed for the whole series of patients and for subgroups stratified by family history of breast cancer, histology, estrogen receptor status, nodal status or tumour grade. Odds ratios for subgroups were based on a comparison with the total control series. A two-sided p value <0.05 was considered to be significant. For a combined evaluation of the three series, stratified analyses were run and Mantel-Haenszel Odds Ratios were calculated using EpiCalc 2000 (www.brixtonhealth.com/epicalc.html).

In addition, overall survival and breast cancer specific survival were analysed for HaBCS cases with available follow-up data (n = 829) with Kaplan Meier statistics using SPSS12 software (SPSS, Munich). Survival endpoints, measured from the date of diagnosis, were any death or death from breast cancer for OS or BCSS, respectively, and alive at last follow-up (censored at 10 years).

Bioinformatic analyses of missense substitutions were performed using the program CONDEL that combines various tools such as SIFT, Polyphen2, and MutationAssessor to calculate the consensus deleteriousness score of non-synonymous variations (http://bg.upf.edu/condel/home). [28]. Possible effects of identified substitutions on splicing were tested using MaxEntScan (http://genes.mit.edu/burgelab/maxent/Xmaxentscan_scoreseq.html) [29]. Global frequency data were available on the Ensembl website of the 1000genomes project (www.1000genomes.org).

### Ethics Statement

Written informed consent was obtained from each patient, and the study was approved by the Ethics Commission of the State Organization “Institute for Hereditary Diseases”, Ministry of Health, Republic of Belarus, by the Ethical Committee of the Institute of Biochemistry and Genetics in Ufa, Bashkortostan, and by the Institutional Review Board at Hannover Medical School, Hannover, Germany (Ethics vote no. 6079, last modification Sep 18, 2012).
